# Modeling the spatio-temporal dynamics of porcine reproductive & respiratory syndrome cases at farm level using geographical distance and pig trade network matrices

**DOI:** 10.1186/s12917-017-1076-6

**Published:** 2017-06-07

**Authors:** Sara Amirpour Haredasht, Dale Polson, Rodger Main, Kyuyoung Lee, Derald Holtkamp, Beatriz Martínez-López

**Affiliations:** 1 Center for Animal Disease Modeling and Surveillance (CADMS), Department of Medicine & Epidemiology, School Veterinary Medicine, University of California, 2108 Tupper Hall, one Shields Avenue, Davis, California, 95616 USA; 2 Boehringer-Ingelheim Vetmedica Inc, Saint Joseph, Missouri USA; 3 Department of Veterinary Diagnostic and Production Animal Medicine, College of Veterinary Medicine, Iowa State University, Ames, USA

**Keywords:** Parameter-driven model, Risk assessment, Bayesian approach, Disease dynamics, Risk-based surveillance, Decision making

## Abstract

**Background:**

Porcine reproductive and respiratory syndrome (PRRS) is one of the most economically devastating infectious diseases for the swine industry. A better understanding of the disease dynamics and the transmission pathways under diverse epidemiological scenarios is a key for the successful PRRS control and elimination in endemic settings. In this paper we used a two step parameter-driven (PD) Bayesian approach to model the spatio-temporal dynamics of PRRS and predict the PRRS status on farm in subsequent time periods in an endemic setting in the US. For such purpose we used information from a production system with 124 pig sites that reported 237 PRRS cases from 2012 to 2015 and from which the pig trade network and geographical location of farms (i.e., distance was used as a proxy of airborne transmission) was available. We estimated five PD models with different weights namely: (i) geographical distance weight which contains the inverse distance between each pair of farms in kilometers, (ii) pig trade weight (*PT*_*ji*_) which contains the absolute number of pig movements between each pair of farms, (iii) the product between the distance weight and the standardized relative pig trade weight, (iv) the product between the standardized distance weight and the standardized relative pig trade weight, and (v) the product of the distance weight and the pig trade weight.

**Results:**

The model that included the pig trade weight matrix provided the best fit to model the dynamics of PRRS cases on a 6-month basis from 2012 to 2015 and was able to predict PRRS outbreaks in the subsequent time period with an area under the ROC curve (AUC) of 0.88 and the accuracy of 85% (105/124).

**Conclusion:**

The result of this study reinforces the importance of pig trade in PRRS transmission in the US. Methods and results of this study may be easily adapted to any production system to characterize the PRRS dynamics under diverse epidemic settings to more timely support decision-making.

## Background

Porcine reproductive and respiratory syndrome virus (PRRSV) is a RNA virus of the family *Arteriviridae* that causes reproductive failure in breeding stock and respiratory disease in piglets. In the US, it has been estimated that the annual economic impact of PRRSV for the pig industry is US$664 million [11]. Previous studies have described three main routes of PRRSV transmission between farms: (i) close contact between pigs, (ii) airborne transmission, particularly in winter and over distances of less than 3 km and, (iii) semen [1]. Spread via semen is relatively easy to control and has been minimized thanks to the increase of biosecurity at boar studs; however transmission through airborne spread or pig movements is more complicated to control as it requires substantial investments and changes in farm management practices (e.g., air filtration, active surveillance, pre-movement testing, etc.). Although the main pathways of transmission between herds have been described, there are few observational studies characterizing the dynamics of PRRS transmission and quantifying the role and relative importance of each pathway in endemic settings. Moreover, very few studies have used information about pig movements and potential airborne transmission (i.e., distance matrices) to forecast PRRS status on farm in an attempt to support the implementation of cost-effective, risk-based interventions. The prevalence of PRRS infection can be high (>40%) particularly in regions with a high density of pigs, large number and frequent pig movements and a lack of vaccination or control measures [1, 9, 14]. However, a better understanding of transmission patterns under specific endemic settings and the prediction of farms at highest risk of PRRS occurrence in subsequent time periods may help producers to prioritize interventions and minimize farm-to-farm transmission, which will facilitate more cost-effective prevention, control and, ultimately, elimination of PRRSV at the farm or regional level.

Mathematical models that quantify the role of each transmission route between farms can be used to better understand the observed distribution of infection as well as predict future outbreaks [5]. Considering the rapid and continuous changes in pig demographics and trade as well as changes in PRRS status and the potential emergence of new diseases (e.g. porcine epidemic diarrhea) there is a need to use models that are flexible to incorporate those changes based on the availability of the data and that allow real-time prediction and modelling of new outbreaks [12].

In this paper, we have expanded and adapted an innovative two-step parameter-driven (PD) approach described by Schrödle et al. [21] which models the spatio-temporal dynamics of PRRS to predict future disease events. The model was computed using integrated nested Laplace approximation (INLA), a recent method for approximate Bayesian inference using latent Gaussian models [19]. INLA is a promising alternative to Markov chain Monte Carlo (MCMC) methods that provides very accurate results within short computational time [20]. We aimed to assess the role that pig trade and geographical distance (as a proxy of airborne transmission) may have on PRRSV transmission and evaluate the predictive ability of PD models to forecast local PRRS cases in a 6 month time frame.

## Methods

### Data

Data on PRRS diagnostic cases, pig demographics and pig movements were provided by pig producers in one US state. The PRRS data comprised 237 reported PRRS cases and more than 56,731 pig movements occurring between >500 production sites from 2012 to 2015. The state, name and exact number of production sites are omitted here to preserve confidentiality. More information about the data set used regarding farm demographics and pig trade network can be found in Lee et al. [15]. Information on the exact geographical location (x, y coordinates) and number of pigs on the farm were also obtained. PRRS surveillance is primarily conducted in sow farms, nurseries and gilt development units (GDUs), as well as in other types of production sites (e.g., finishers and wean-to-finish farm; WF) Therefore, after an outbreak investigation, we focused only on modeling PRRS cases in sow farms, nurseries and GDU, and the subpopulation of finishers and wean-to-finisher farms that had reported PRRS outbreaks in the last 4 years. In summary a total of 124 farms were considered for the analyses.

### Modelling approach

We used a two step approach. First, we used a parameter driven model to identify if there is a spatio-temporal trend of PRRS reported cases from Jan 2012 to July 2014. Then, if a spatio-temporal trend existed, an autoregressive parameter driven model was used to predict the PRRS cases for the last half of 2014 based on different weight matrices. All the analyses were done by using R-INLA software package for R ([19]; www.r-inla.org).

### Parameter driven model

We assumed that the general binary observation of PRRS outbreaks in farm *i* at time *t* (*Y*_*it*_) follows a Binomial distribution of the form:

1 $$ {Y}_{it}\kern0.1em \sim \kern0.1em \mathrm{B}\mathrm{i}\mathrm{n}\mathrm{o}\mathrm{m}\mathrm{i}\mathrm{a}\mathrm{l}\kern0.1em \left({m}_{it},\kern0.5em {\eta}_{it}\right)\kern0.1em \mathrm{w}\mathrm{i}\mathrm{t}\mathrm{h} \hspace{0.1em} t=1,2,\dots, T; i=1,2,\dots, I $$

where the number of trials, *m*_*it*_, adjusts for possible numbers of tested individuals on farm. In this study we assumed 10% of the animals (i.e. number of trials) in each farm were being tested in a 6-month period. Therefore the probability that the farm is having a outbreak based on the PRRS test result is following binomial distribution. Here *T* is equal to six corresponding to the number of 6-month time steps from 2012 to 2015, *I* is equal to 124 (i.e. number of the farms in this study) and *η*_*it*_ is the probability of having at least one positive animal in the farm, which is specified as recommended by Blangiardo and Cameletti, [3]:

2 $$ l o g i t\left({\eta}_{i t}\right)={b}_0+{u}_i+{\nu}_i+\left(\kappa +{\delta}_i\right) t $$

where *b*_0_ is the intercept, which quantifies the average PRRS farm status in the entire study, while *u*_*i*_ and *ν*_*i*_ are the area-specific effects. The parameter *u*_*i*_ is assumed to be structured in space, which takes into account the PRRS status in neighboring farms [18]. We defined neighboring farms as one located within 6 km radius.

Conditional autoregressive [2], was specified as the structure for the ***u*** = {*u*_1_ *,*…*, u*_*n*_}. Considering *n* areas, each characterized by a set of neighbors ${\mathcal{N}}_i$ and assuming *u*_*i*_ is the following random variable [3]:

3 $$ {u}_i\mid \boldsymbol{u}- i\sim Normal\left({\mu}_i+\sum_{j=1}^n{r}_{i j}\left({u}_j-{\mu}_j\right),{s}_i^2\right) $$

where *μ*_*i*_ is the mean for the farm *i* and ${s}_i^2={\sigma}_u^2/{\mathcal{N}}_i$ is the variance for the same farm, which depends on its number of neighbors (e.g., if an farm has many neighbors then its variance will be smaller). This variance structure recognizes the fact that in the presence of strong spatial correlation, the more neighbors a farm has the more information there is in the data about the value of its random effect. While the variance parameter ${\sigma}_u^2$ controls the amount of variation between the spatially structured random effects. The quantity *r*_*ij*_ indicates the spatial proximity and can be calculated as *ϕ* ×*W*_*ij*_, where *W*_*ij*_ = ${a}_{i j}/{\mathcal{N}}_i$, *a*_*ij*_ is 1 if farms *i* and *j* are neighbors and 0 otherwise (note that *a*_*ii*_ is set to 0, thus *W*_*ii*_ and *r*_*ii*_ are 0 as well); and finally, the parameter *ϕ* controls the properness of the distribution as it was formulated by Cressie [4].

The parameter *ν*_*i*_ is a spatially unstructured component which follows a Normal distribution of the form$\mathrm{N}\left(0,{\ \sigma}_{\nu}^2\right)$; where ${\sigma}_{\nu}^2$ is the variance of the marginal unstructured component. The main linear trend *κ*, represents the global time effect. A differential trend *δ*_*i*_, which identifies the interaction between time and space, represents the difference between the global trend (*κ*) and the area specific trend. If *δ*_*i*_ < 0 then the area specific trend is less steep than the mean trend, whilst *δ*_*i*_ > 0 implies that the area specific trend is steeper than the mean trend.

### Autoregressive parameter driven model

For this step we used a hierarchical Bayesian model similar to the one described by Schrödle et al. [21]. Equation 2 can be re-written using two stages.

4 $$ \begin{array}{l}\mathrm{Stage}\ 1:\mathrm{logit}\left(\ {\eta}_{i t}\right)={b}_0+{u}_i+{\nu}_i+\left(\kappa +{\delta}_i\right) t+{\zeta}_{i t}\\ {}\mathrm{Stage}\ 2:{\zeta}_{i t}=\lambda .{\zeta}_{i, t-1}+\rho {\sum}_{j\ne i}{\omega}_{j i}.{\zeta}_{j, t-1}+{\epsilon}_{i t}\end{array} $$

Because PRRS spread follows more an endemic than epidemic pattern in our particular study area *u*_*i*_ and *ν*_*i*_ are included in stage one as suggested by [21].

Equation 3 includes an autoregressive process *ζ*_*i*_ = (*ζ*_*i*1_,  … , *ζ*_*iT*_)^*T*^ for each farm *i* to model the latent spatial spread of the disease based on the georeferenced location of the farms (e.g. [3]). In the second stage, λ and *ρ* are the autoregressive parameters. We used N(0, 0.25) distributions as priors for *λ* and *ρ* in all the models. The term $\sum_{j\ne i}{\omega}_{j i}.{\zeta}_{j, t-1}$ is a weighted sum of the past states on other farms *j* different than the farm of interest (*i*). Different choices for the weights *ω*_*ij*_ are possible (e.g., [16]). Here we used five different weights namely: (i) geographical distance weight which contains only the inverse distance (1/*i* ~ *j*) between each pair of farms in kilometers, (ii) pig trade (*PT*_*ji*_) weight which contains the number of pig shipments between each pair of farms and, (iii) the product between the distance weight and the standardize relative pig trade weight (*PT*_*ji*_/number of animals in sender farm), (iv) the product between the standardized distance weight (standardized distance (*i* ~ *j*) between each pair of farms in kilometers) and the standardized relative pig trade weight (standardize pig trade (*PT*_*ji*_) weight/ number of animals in sender farm), and (v) the product of the distance weight and the pig trade weight. For the geographical distance weight the matrix is symmetric. Other combinations using pig trade weight and distance weight in the denominator were tried with but no convergence was obtained. However, the pig trade weight is not symmetric for each pair of farms, because the number of pig traded from farm *j* to farm *i* in our study was different from the number of pig traded from farm *i* to farm *j*. The errors *ϵ*_*i*_ = (*ϵ*_*i*1_,  … , *ϵ*_*iT*_)^*T*^ were assumed to be independent and normally distributed with variance${\sigma}_{\epsilon}^2$.

We calculated the mean of the first (finite) moment of the predictive probability distribution (*μP* = *E*(*y*_*iT*_ | *y* − *T* ) as defined by Schrödle et al. [21]. Here, the vector *y* − *T* contains all the observations in all regions *i* up to time *T*-1.We used *μP* to categorize the PRRS status of farms into two groups namely negative and positive (i.e., farm predicted as negative or positive by the model). The optimal threshold based on the *μP* was calculated using the true positive rate (sensitivity) and true negative rate (specificity). The optimal threshold is the threshold that maximizes the distance to the identity line in the ROC curve [24].

### Model selection criteria and prediction ability

We used the deviance information criteria (DIC), which can be easily calculated in INLA, as the selection criterion for the best final Bayesian model [22]. Smaller DIC values indicate a better trade-off between complexity and fit.

In order to determine the discriminating power for distinguishing between PRRS negative and positive farms we calculated the area under the Receiver Operating Characteristic (ROC) curve (AUC) based on the calculated the mean of the first (finite) moment of the predictive probability distribution (*μP*) of each model [8]. The higher the AUC, the better the model performed in predicting the infected and not infected farms in the last half of 2014. We also computed the simulation error and the model prediction error. The simulation error is the number of times in which the PRRS farm status is incorrectly classified based on the calculated *μP* from 2012- first half of 2014 (5 time steps). The model prediction error is the sum of the false positive farms and the false negative farms based on the calculated *μP* of the second half of 2014.

## Results

**Fig. 1:**
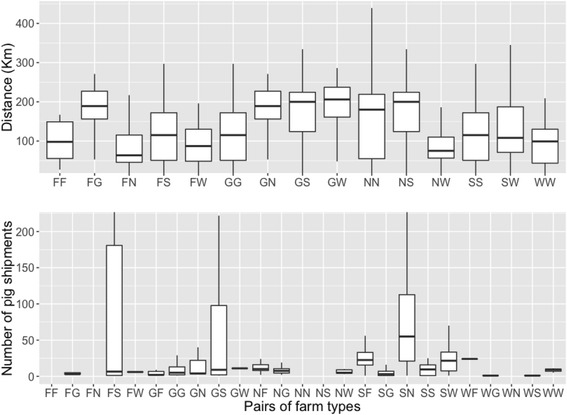
Boxplots illustrating the distance (*above*) and number of pig shipments (*below*) between pairs of farm types (where F, G, N, S and W stands for finisher, gilt development units, nursery, sow farm and wean-to-finish, respectively). The first letter indicates the origin of the pigs. Notice that only a small fraction of finishers and wean-to-finish were included in the analyses (i.e., only the ones with PRRS outbreaks) therefore movements from and to those farms are not representative of the total volume of movements in the entire population. The horizontal axis (i.e., pairs of farm types) differs in both charts as the pairwise comparison in distance is symmetric but it is asymmetric in movements (i.e., pig movements FG ≠ GF)

A summary of the distance and number of pig shipments between different types of farms is shown in Fig. 1. The average distance between the farms was 135.4 km. There were 88 pairs of farms out of 7564 pairs of farms that were located in less than 6 km from each other. Most of the shipments (81%) originated from GDUs.

**Fig. 2:**
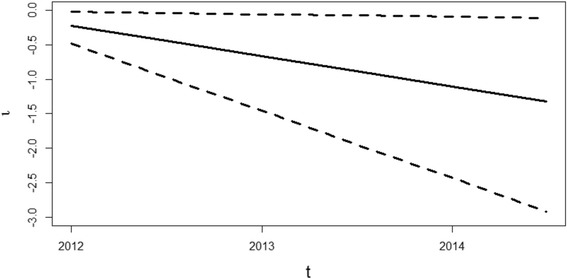
Global linear temporal trend for PRRS cases from January 2012 to July 2014 using a time step of 6 months. The solid line identifies the posterior mean for *ι*_*t*_ while the dashed lines are 95% credibility interval

**Fig. 3:**
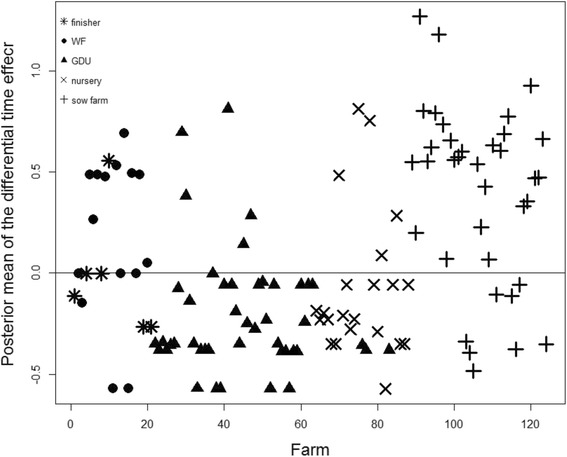
Posterior mean of the differential time effect (*δ*_*i*_) for reported PRRS cases of 124 farms from 2012 to 2015. If *δ*_*i*_ < 0 then the farm specific trend is less steep than the mean trend, whilst *δ*_*i*_ > 0 implies that the farm specific trend is steeper than the mean trend. Star, Circle, triangle, multiply and plus represent finisher, WF, GDU, nursery and sow farm, respectively

Results of the spatio-temporal parameter driven model of the PRRS status on farm are shown in Figs. 2 and 3. A downward sloping trend in the risk of PRRS cases over time is apparent when plotting the *μP* of the main time effect (*κ*_*t*_) together with its 95% credibility interval (Fig. 2). By plotting the *μP* of the differential time effect (*δ*_*i*_) we observed that the differential temporal trend is below the average mostly in the GDU and nursery, while the differential temporal trend for the other site types were higher than the average (Fig. 3).

The fit and predictive performance of the different autoregressive PD models (equation 3) are indicated by DIC and AUC (Table 1). The AUC value for the model using only the geographical distance weight had a lower AUC (0.83) compared with the other models suggesting a lower predictive ability. The AUC value of the models using relative pig trade rate combined with distance weight was lower (AUC = 0.86 and AUC = 0.78) than the model using only the pig trade weight matrix (AUC = 0.88). The model using pig trade weight matrix was also the best fit according to the DIC.

**Table 1:** The area under the ROC curve (AUC) based on the calculated posterior mean for the PRRS outbreaks in the farms of second half of 2014 for autoregressive parameter driven model and calculated deviance information criteria (DIC). “std” is the abbreviation for standardize matrix

*W*_*ji*_	AUC	DIC
1/*i* ~ *j*	0.83	−5540.065
*PT*_*ji*_	0.88	−5382.211
(*PT*_*ji*_ /*m*_*i*_) . *i* ~ *j*	0.86	NA^a^
(std*PT*_*ji*_ /*m*_*i*_) . std*i* ~ *j*	0.78	−5540.059
*PT*_*ji*_ . *i* ~ *j*	Not converged	Not converged

^a^R-Inla was not able to calculate the DIC for this model

**Fig. 4:**
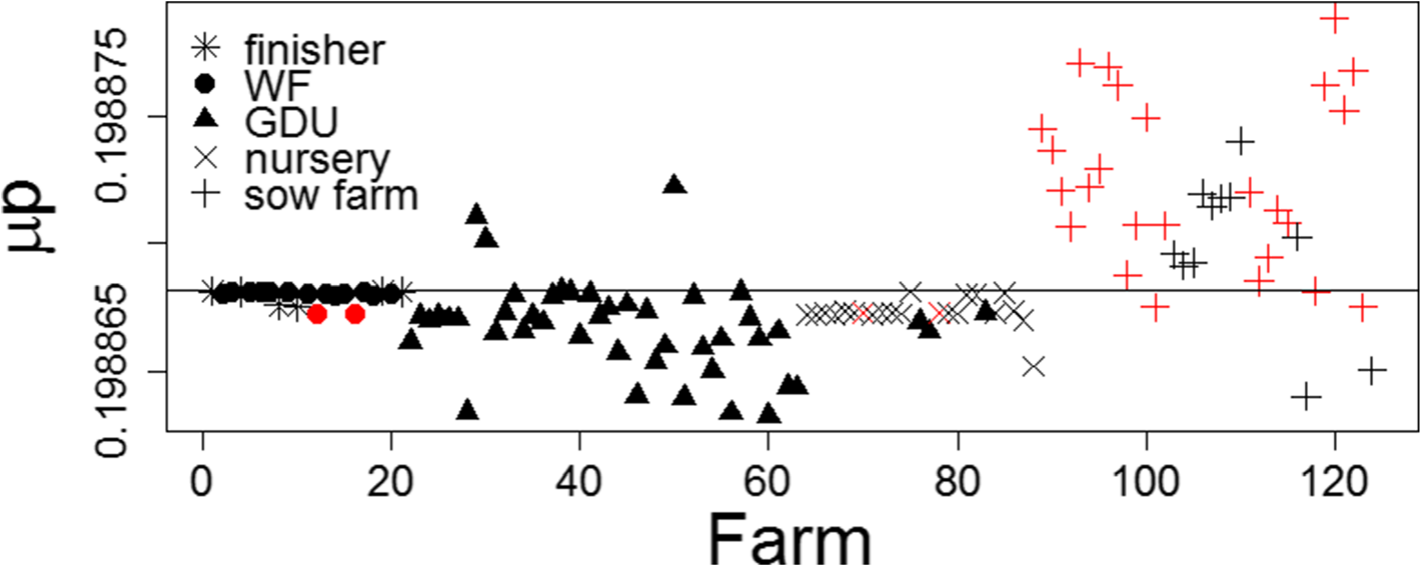
Posterior mean (*μP*) for reported PRRS cases of 124 farms for the last half of 2014. The farms below the horizontal line (threshold) are predicted negative and the one above the horizontal line are predicted positive. The reported positive farms in the second half of 2014 are *red* whilst the reported negative farms are *black*. The model prediction accuracy is the sum of the black farms below the threshold and the red farms above the threshold divided by total number of farms (105/124)

The calculated threshold to categorize the farms into two groups based on the *μP* has the value of 0.1988682. The prediction accuracy of the model based on the defined threshold was 85% (105/124) (Fig. 4).

**Fig. 5:**
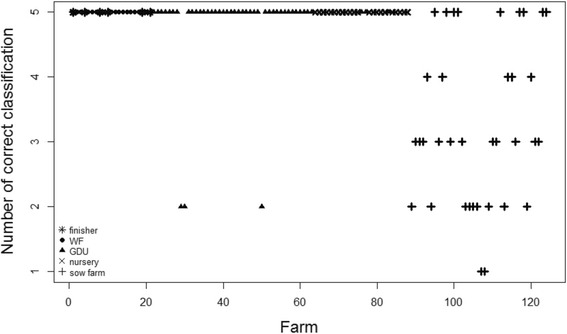
The number of simulations in which the status of the farm was correctly classified as positive or negative based on the calculated posterior mean from 2012- first half of 2014 (5 time steps)

Figure 5 shows how the model performs to simulate the PRRS cases in 124 farms from 2012- first half of 2014 (5 time steps). The model correctly simulated the PRRS status in most of the farms in each of the five time steps except in 27 of the sow farms and three GDU farms.

## Discussion

In this paper, we used an advanced two-step parameter-driven approach to evaluate the spatio-temporal dynamics of PRRS from 2012 to July 2014 and predict the subsequent farm status (second half of 2014) in 124 swine production sites of US. The model using the number of pig moved between the farms as a weight matrix exhibit a better predictive performance than the model just assuming the airborne spread from nearby farms. This suggests that pig trade may have a more important role in PRRS transmission dynamics than airborne transmission, at least in the endemic setting evaluated here. This model was also able to provide a temporal trend for the system (Fig. 2), which may be used to visualize if a system is improving over time and individual farm “scores” (Fig. 3) that could be used for benchmarking and prioritization of risk-reduction measures.

Throughout the global swine industry, extensive efforts have been made to protect commercial swine farms from infection with PRRSV. Extensive efforts are directed to prevent the entry of virus via indirect routes of transmission such as contaminated transport vehicles and insects or aerosols [6, 7]. [6] evaluate the potential of PRRSV-contaminated transport vehicles to infect naïve pigs and assess four sanitation programs for the prevention of virus spread. However, their results support the claim that the movements of animals between farms may play a more important role in the transmission of PRRSV between farms than the nearby airborne transmission of virus.

Previous studies have demonstrated the value of using spatio-temporal models to unravel disease dynamics and identify high risk areas for disease occurrence. To the best of our knowledge very few models have been published to predict PRRS status in the US swine industry. A study conducted by [17] in Ontario, Canada investigated evidence of spread of PRRSV genotypes and determined if spread could be attributed to supplier or ownership connections between herds. The spatial and temporal distributions of six PRRSV genotypes were investigated from 2004 to 2007. Their investigation found no strong evidence of PRRSV spread via aerosol between pig herds in Ontario. They conclude that cluster analysis might not be sensitive for real-time surveillance of PRRSV outbreaks and pig movement network information was more important in the spatial spread of the PRRSV. Kwong et al. [13] examined spatial and temporal spread of PRRSV using individual-level models (ILMs) for infectious diseases, fitted in a Bayesian MCMC framework. Their results showed that the three most important factors for the spread of PRRSV in Ontario (Canada) swine herds were sharing the same herd ownership, gilt source and market trucks. Kwong also suggested that spatial proximity could not be identified as important contributor to PRRSV spread. The results presented in our study provides further evidence that animal movements may be playing a more important role in PRRSV transmission than previously thought in PRRSV endemic settings. Moreover, we have demonstrated the value of using this information to predict, with relatively high accuracy (i.e., 87%) the PRRSV status on farm in subsequent time periods. The model presented here provides a simple, convenient framework and can be employed in real-time using data on the underlying population at risk (i.e., herd size), the PRRS status of farms, the geographical location of farms and, the pig trade network. All this data is usually available for most commercial US pig production systems. Hence our approach could easily be implemented in operational, real-time surveillance, risk assessment and modeling systems such as Disease BioPortal™ (http://bioportal.ucdavis.edu) to support daily decision making for producers and associated stakeholders.

Most of the prediction errors (11 out of 17 errors in Fig. 4) and all the simulation errors (Fig. 5) occurred in sow farms. The majority of the prediction errors in the sow farms are false positive (seven false positive and four false negative). This result may be associated to some underreporting of PRRS cases in sow farms or to the epidemiological impact and changes in surveillance associated to the introduction and the massive spread of porcine epidemic diarrhea virus (PEDV) during 2013 in the US. PEDV caused significant production losses in the swine industry [23]. The PEDV epidemic also led to an increase of biosecurity measures, disruption of pig trade and reduced sampling and diagnosis of PRRSV [10], which may explain the significant reduction of PRRS reported cases in 2013. Unfortunately, information about biosecurity measures or PED reported cases on farm were not available at the time of this study. The consideration of such information may help to explain some of the simulation and prediction error and potentially increase the predictive accuracy of the model if biosecurity is included as part of the weight matrix.

Another limitation of this study is that PD model does not allow formulating the probability of having at least one positive animal in the farm (i.e., probability that a farm is positive) using multiple weight matrices. However, the weight matrix can be formulated to incorporate different covariates or as a function of different weight matrices to remedy this problem, as it is suggested by Schrödle et al., [21] and as we did in this study (although some of those combinations unfortunately did not converge (Table 1). However, the use of combined weighted matrices limit us in understanding the impact of the each weight matrices and their impact on the probability that a farm is PRRS positive. In this paper we combined the distance weight matrix with the pig trade weight matrix in three different formulations based on the suggestions from Schrödle et al. [21] (Table 1). But none of them had better predictive ability than the model considering only the number of pig movements between farms. One of the reasons why the model using the distance matrix combined with the pig trade weight does not have a better predictive ability than the model using the pig trade weight alone may be that only 1.1% (88/7564*100) of the pair of farms in this study is located less than 6 km from each other. Moreover, farms belonging to other systems that could be near our study farms were excluded from the analyses. Further studies are needed to evaluate if similar results are obtained in higher density areas with more farms within the 6 km range and including other pig production systems.

## Conclusion

This study provides a convenient data-driven modelling approach easy to implement and update to characterize PRRS spatio-temporal transmission dynamics and to accurately model PRRS status on farm for subsequent time periods. Model results reveal that pig trade is the most important pathway contributing to PRRSV transmission between farms in the endemic setting under study. A better understanding of pig trade networks and the use of models like the one presented here can help to better predict and control future PRRS outbreaks in US and other endemic settings. These methods will be integrated within the Disease BioPortal™ (http://bioportal.ucdavis.edu) to facilitate the daily use and operational long-term availability of these tools.

## Abbreviations

AUC: Area Under the ROC Curve
DIC: Deviance Information Criteria
INLA: Integrated Nested Laplace Approximation
PD: Parameter-Driven
PED: Porcine Epidemic Diarrhea
PEDV: Porcine Epidemic Diarrhea Virus
PRRS: Porcine Reproductive and Respiratory Syndrome
PRRSV: Porcine Reproductive and Respiratory Syndrome Virus
ROC: Receiver Operating Characteristic
